# A Retrospective Study on the Outcomes of Injuries From Border Wall Falls

**DOI:** 10.7759/cureus.57411

**Published:** 2024-04-01

**Authors:** Sharmeen Azad, Andrew McCague, Austin Henken-Siefken

**Affiliations:** 1 Surgery, Desert Regional Medical Center, Palm Springs, USA; 2 Surgery, Western University of Health Sciences, Lebanon, USA; 3 Trauma and Acute Care Surgery, Desert Regional Medical Center, Palm Springs, USA

**Keywords:** immigration, falls, injuries from falls, mexican border wall, border wall fall

## Abstract

Objective

Our retrospective cohort study focuses on the outcomes of injuries sustained from falls from the USA-Mexico border wall. The purpose of this study is to understand and predict the types of injuries that will be present in patients who fall from the border wall. This can further help trauma response teams to better predict and prepare for the care of these patients.

Methods

This retrospective cohort study included all patients that were admitted to Desert Regional Medical Center, a trauma I center, after a fall from the border wall that ranged from heights of 15 to 30 feet. The admissions occurred between March 2016 to December 2021.

Results

Of the 108 patients included, 38.2% (78) sustained at least one lower extremity injury, of which the most common was injury to the calcaneus bone. Additionally, there were several concomitant injuries, of which the combination of lower extremity and lumbar injury was found to be the most common (11.2%). The injury severity score (ISS) was found to not be statistically significant (ɑ=0.05) between groups of patients whose length of stay (LOS) in the hospital was greater than 10 days and less than 10 days. There was 1% fatality (1 of 108) and 92.5% required surgical intervention (100 of 108).

Conclusions

Patients injured from border wall falls are more likely to sustain lower extremity injuries than injuries to other parts of the body. Additionally, patients with lower extremity injuries sustained lumbar spinal injuries concomitantly, which can be most likely attributed to the axial compression of the spine during these falls. Most of these injuries required surgery and hospital admissions to treat. Understanding the patterns of injury from border wall falls can further help trauma response teams treat patients with efficient management.

## Introduction

For many years, migrants have risked their lives to cross the US-Mexican border in the hopes of beginning a better life that is free of war and violence. However, the risk of crossing the border has only increased with time. A considerable share of admissions to trauma hospitals in border areas are due to injuries experienced from jumps or falls along the US-Mexico border [1]. These falls result in traumatic injuries with life-long consequences. As the height of the border wall increased during the Trump administration, these falls have shown to be more life-threatening with injuries that have also created significant financial burdens on hospitals [2]. 

Despite the rise in the number of falls in recent years, little is known about the types of injuries sustained and the general outcomes of these falls. Some studies have shown that spinal and extremity injuries caused by falls near border walls were far more frequent than cranial injuries [3]. However, our study focuses on the primary and concomitant injuries sustained during these falls. Our retrospective study included 108 patients who were admitted to Desert Regional Medical Center, a level I trauma center, for border wall falls between March 2016 and December 2021. These patients were deidentified and data on their admission was collected. This included date of admission, gender, age, sex, injury description, surgery and ICU status. We aimed to focus on the types of injuries most patients sustained. This information will be advantageous for trauma centers near the US-Mexican border to better prepare for the care of these patients. 

## Materials and methods

This Institutional Review Board (IRB)-approved (MetroWest IRB, approval 2020-111) retrospective cohort study included patients who were admitted after a fall from the US-Mexican border wall, which ranged from heights of 15 to 30 feet, to Desert Regional Medical Center between March 2016 to December 2021. This database only contained information from inpatient admissions to the hospital from the emergency department. Cases involving border wall-related falls included a total of 108 admissions (n=108). A border wall fall injury was defined as an injury that occurred as a direct result of a fall from the border wall while attempting to cross the border. Deidentified data collected on the patients from the trauma registry included age, sex, injury severity score (ISS), Glasgow Coma Scale (GCS), hospital length of stay (LOS), surgery status and ICU admission. The patterns of injury were collected and analyzed. Variables were initially investigated using descriptive statistics. Variables investigated included injury event details (date, time, place), patient demographics (age, gender) and injury details (type and location). Injury events with multiple incident types were also noted.

To explore the effect of incident type on lower extremity injury, incident type, as well as any variables that were found to be significant during univariate regression exploration, was entered into a multivariate binary logistic regression. Whether an injury event involved multiple incident types (yes/no) was also included due to previously found association with severity of injury. Statistical analysis was completed with Microsoft Excel™ (Redmond, WA, USA). All tests were 2-sided with ɑ = 0.05. The continuous variables were compared using the independent samples t-test. We collected data from the de-identified database and reviewed the descriptions and diagnosis to determine the types of injuries sustained. We did all of the calculations from the data using Microsoft Excel™. 

## Results

There were a total of 108 patients in the database. Table 1 shows that the median age of the patients was 28.5, with a standard deviation of 9.56. Males comprised 69.4% (N = 75) of our patients, while 30.6% (N = 33) were female. 98.1% (N = 106) of the patients were admitted to the hospital, with 16.7% (N = 18) admitted to the ICU, 2.8% (N = 3) requiring ventilation and 93.5% (N = 101) requiring surgery.

**Table 1:** Injury Severity Score by Length of Stay (LOS)

	LOS < 10 days	LOS >10 days
N = number of patients	66	42
Mean (+SD)	8.98 (+5.60)	10.43 (+6.92)
Median	9	9
P-value	0.26	

We further assessed injury type by body region, as shown in Table 2. The data shows that 38.2% (N = 78/204) of the injuries sustained were lower extremity injuries, of which the most common types included calcaneus fractures or displacement. Other lower extremity injuries included tibial, fibular, and navicular fractures and displacements. The second most common injury seen was lumbar spine (N = 27, 13.2%) which mostly included fractures. The third most common injury included head and neck injuries (N = 26, 12.7%), characterized by unspecified injuries of the head, hemorrhagic contusions, and abrasions. Upper extremity (N = 19, 9.3%), thorax (N = 17, 8.3%), pelvis/sacral (N = 15, 7.4%), thoracic spine (N = 12, 5.9%), and abdomen (N = 10, 4.9%) injuries were shown to be the least common. 

**Table 2:** Injury type by body region

Body Region	Number of injuries: N = 204	Percentage %
Head/Neck	26	12.7%
Thorax	17	8.3%
Thoracic Spine	12	5.9%
Lumbar spine	27	13.2%
Abdomen	10	4.9%
Pelvis/Sacrum	15	7.4%
Upper Extremities	19	9.3%
Lower Extremities	78	38.2%

We assessed the average ISS in comparison to the average LOS of patients in the hospital (Table 3). The results show that the median ISS score for all patients was 9. The mean for those with LOS less than 10 days was 8.98 days and for those who stayed longer than 10 days was 10.43. The standard deviation in patients who were hospitalized for less than 10 days was 5.6 and the standard deviation for those hospitalized for more than 10 days was 6.92. We did a two-tailed paired t-test analysis on these two groups using Microsoft Excel™ and found that the p-value was 0.26, showing no statistical significance between the two groups, indicating that there is no association between ISS score severity and length of hospital stay.

**Table 3:** Injury Severity Score by Length of Stay (LOS)

	LOS < 10 days	LOS >10 days
N = number of patients	66	42
Mean (+SD)	8.98 (+5.60)	10.43 (+6.92)
Median	9	9
P-value	0.26	

We further analyzed the concurrence of other injuries with lower extremity injuries. There were a total of 78 lower extremity injuries. The majority of patients who had lower extremity injuries, had so without concomitant injuries (48.7%, N = 35). Of those who had concomitant injuries, we found that lumbar spinal injuries consisted of 11.5% (N = 9) of the injuries, making it the the most common single co-occurring injury with a lower extremity injury (Table 4). Two or more different body injuries in addition to their lower extremity injuries comprised 23% of the lower extremity injuries (N = 18), head & neck and upper extremity injuries comprised 6.4% of the injuries (N = 5), whereas thorax, abdomen and pelvis all comprised 1.3% (N = 1) of the injuries each. 

**Table 4:** Lower extremity injuries with concomitant injuries

Lower Extremity (LE) + Other injury	Number of patients with multiple injuries (N) = 78	Percentage % (out of 78 total concomitant)
Total	78	100%
LE+ Head & Neck	5	6.4%
LE + Thorax	1	1.3%
LE + Abdomen	1	1.3%
LE + Pelvis	1	1.3%
LE + Upper Extremity	5	6.5%
LE + Lumbar Spine	9	11.5%
LE+ Two or more regions	18	23%
Total concomitant	40	51.2%

## Discussion

In our retrospective study of 108 cases of falls from the Mexico-US border wall, we found that there were many significant injuries caused by these falls. The most common body region injury sustained was lower extremity injuries, followed by lumbar injuries. Of the 108 patients, one patient died and 101 required surgical intervention. Previous research supports our conclusion that in border wall falls, there is a much higher predominance of lower extremity injuries [1,2]. Recent studies have associated the increased border height with falls resulting in more morbid and costly extremity, spine, and intracranial injuries [4,5]. We further analyzed this and found that the most common occurrence of concomitant injuries with lower extremities injuries are lumbar spine injuries.

In patients with lower extremity injuries, we saw a pattern of displaced open tibial and fibular fractures and these were primarily treated surgically. Thoracic and lumbar spine injuries consisted of wedge compression fractures, especially in the second, third and fourth vertebrates and upper extremity injuries consisted mostly of closed radial and ulnar fractures.

We performed a statistical analysis using Microsoft Excel™, in which we determined that length of stay in the hospital and the injury severity score was not statistically significant (ɑ = 0.05) between patients who stayed in the hospital for less than 10 days and patients who stayed for greater than 10 days. A possible cause for this result can be due to the issue of disposition [6]. Some patients with a lower ISS score stayed in the hospital for more days than needed as they had no place to go after discharge. Additionally, many facilities that these patients would normally be designated to go to after discharge, such as rehab or nursing homes, refused to accept the migrants. This left many patients stranded in the hospital for a much longer time.

Although our study showed that lower extremity injuries are the most common injuries sustained from border wall falls, there were some limiting factors to our study. Our sample size was relatively small (n=108), which may not have captured the overall percentage of the population. Thus, our data may not be fully representative of all falls from the border wall. We also do not have data on the patients who had minor injuries or patients who died on the scene due to their injuries. An additional limitation of our study is the lack of information in the database about the specific treatments patients received for their injuries, which would be beneficial for a follow-up study to determine the resources used for these injuries. Our study shows that lower extremity injuries were heavily outweighed by other types of injuries, followed by lumbar spine injuries and head/neck injuries. Previous studies have shown that intracranial injuries occurred less frequently than spine injuries [7], supporting our results. This information may be useful for trauma centers near the border to help optimize preparation for the assessment and evaluation of these patients.

Future considerations for this topic should assess the outcomes of border wall injuries before the border wall height was increased and after the height increased in December 2019. Previous research focused on whether or not constructing the border wall is associated with increased mortality, as described by Bruch [8]. In this paper, it was found that areas where border wall construction was occurring did not have a statistically different change in mortality than areas where there was no border wall construction [8]. Although this may be the case, it was a singular study, so it can still be predicted that the increase in the wall height can contribute to worse injuries, and perhaps even more fatality than presented in our study [5,9-11]. Additionally, future research can focus on the differences in injury type depending on the height of the wall that patients fall from. Research described by Marshal found that the increased height of the US-Mexico border wall has resulted in record numbers of injured migrant patients, placing novel financial and resource burdens on already stressed trauma systems, supporting our findings [5]. Further research into these topics can help contribute to the preparation of trauma centers for these cases.

## Conclusions

Thousands of migrants cross the US-Mexico border each year hoping to find a safe haven in the United States. Many of these migrants suffer significant injuries in the process. With the rise in border wall falls after changes to border wall regulations, it is important to understand the most common types of injuries that trauma centers are seeing. Our study on patients with injuries due to border wall falls has shown that a majority of the patients sustained lower extremity injuries. Of those who sustained lower extremity injuries, more than half also sustained concomitant injuries, with lumbar injuries being the most common. The concomitant nature of these injuries can be attributed to the axial compression that the spine experiences during these falls from various heights. Most of these injuries required surgery and hospital stay to treat. In the midst of political regulations, it is crucial to understand the patterns of injuries from border wall falls to further help healthcare teams treat these patients effectively. 
